# ‘I want every minute to be worthwhile now’: The views and experiences of people living with dementia and their care partners about returning to in-person group meetings after COVID-19 lockdown restrictions

**DOI:** 10.1177/14713012221118768

**Published:** 2022-08-16

**Authors:** Siobhán Kelly, Sophie Bushell, Anthea Innes

**Affiliations:** School of Health and Society, 7046 University of Salford, Salford, UK; Salford Institute for Dementia, 7046 University of Salford, Salford, UK; Salford Institute for Dementia, 13995 University of Salford, Salford, UK

**Keywords:** Covid-19, group meetings, risk, autonomy, social support

## Abstract

COVID-19 and the resulting limitations on freedom of movement has been difficult for many, including individuals living with dementia and those who provide support and care. In the summer of 2021, England’s national lockdown measures eased, and regulations were amended to allow indoor social gatherings. With this enabling a return to in-person meetings, this study explored the experiences of people living with dementia and current and former care partners who had previously attended groups at Salford Institute for Dementia (UK). Two phases of research were conducted. In the first phase, during the summer of 2020, telephone interviews were utilised to ask participants (*n* = 13) about their views of re-engagement and how the in-person groups might be best reintroduced. Phase two began in the summer of 2021, where mood questionnaires (*n* = 10) were administered and observations conducted to explore how participants experienced the return to in-person meetings. Thematic analysis resulted in the construction of three overarching themes: planning for and the reality of transitioning; safety versus autonomy; and tensions and complexities of life in the ‘new normal’. Despite initial concerns about their reintegration into the community, participants all enjoyed resuming in-person meetings. An inclusive and consultative approach to re-engagement allowed all participants to feel valued, safe, and informed about their return to campus. However, individuals living with dementia and care partners experienced the transition to re-engagement in different ways and their perceptions shifted over time. We therefore highlight the complexities of responding to different perceptions of risk and safety, while also promoting engagement and inclusivity after a period of social isolation. In this paper, we consider implications for the re-integration of individuals with dementia and their care partners into in-person social groups and propose further avenues for research.

## Introduction

Living well with dementia is supported by policy (Department of Health & Social Care, 2015) and requires access to mental, physical and social engagement opportunities (Alzheimer Society, 2013). Social groups for people living with dementia in the community can promote wellbeing through fostering a sense of belonging, creating a sense of achievement and purpose, providing opportunities for enjoyable and stimulating engagement and supporting positive and reciprocal relationships (Innes et al., 2021, Morris et al., 2021). However, the measures introduced by the UK Government in March 2020, and to varying degrees around the world, to reduce individuals’ social contact and limit the spread of COVID-19, led to community groups closing their doors and the reduction of vital support networks.

Numerous laws were made in the UK in response to the pandemic. More specifically, England’s national COVID-19 response included: an initial lockdown in March–June 2020; a second lockdown in November 2020; and a third in January–March 2021. Social restrictions were put in place throughout this period to reduce potential transmission, such as limits on household mixing and indoor gatherings, and in March 2021 an official phased exit from lockdown – known as the ‘roadmap out of lockdown’ – was introduced (see Brown and Kirk-Wade, 2021).

Lockdown measures were predicted to have a disproportionate effect on older people within the community and particularly those with cognitive or physical impairment or significant caring responsibilities (Korczyn, 2020). Alzheimer Europe (2020), for instance, forecast that the associated loss of social contact will increase anxiety, depression and stress amongst people living with dementia in the community. Coupled with reports of concerns around a rapid loss of cognitive ability (Rochford-Brennan & Keogh, 2020) as a result of social distancing, as well as the closure of most care and support services (Centre for Ageing Better, 2020), significant concerns have been raised around the impact of lockdown measures on the wellbeing of people living with dementia and their care partners.

Virtual and online meetings offered a way to try and compensate for the lack of in-person social contact and support, which led to a fast-moving shift to the use of digital technology to both keep in touch with loved ones (The Health Foundation, 2020) and deliver health and social care services (Cuffaro et al, 2020; Goodman-Casanova et al, 2020). With access to online social engagement and services requiring a person to have the appropriate technology and the skills to engage with online platforms, advancing age and decreased cognitive ability have been cited as the biggest predictor of reduced digital engagement (Centre for Ageing Better, 2020). This meaning, people living with dementia were among those at the greatest risk of being left behind and experiencing isolation in the current technological revolution (Rochford-Brennan & Keogh, 2020).

It is vital to investigate older individuals social experiences during the pandemic and explore the impact that COVID-19 and consequential social distancingmeasures have had on this group (British Geriatrics Society, 2020) is vital knowledge as in-person meetings begin again. Research reporting on experiences of lockdown has demonstrated the physical impact for people living with dementia (Greenberg et al., 2020) as well as the negative impact on social wellbeing (Roach et al, 2021; Bushell, Innes and Smith forthcoming). Roach et al. (2021) concluded that social re-integration was an area requiring careful consideration. As such this paper contributes to how social re-integration following lockdown was achieved for participants living with dementia and care partners who had previously attended a variety of in-person groups designed to promote social health and wellbeing. We share how the process of planning and beginning to meet in-person was approached after a 15-month period of virtual communications and support and the views and experiences of group members as they met in-person again.

## Methods

Full ethical approval was obtained from the University of Salford ethics panel (approval number phase one: HSR1920-099; approval number phase two: 2153). Research participants were known to the researchers via an initiative known as the Dementia Associates Panel (Innes et al., 2021). The Dementia Associates Panel, based at Salford Institute for Dementia (UK), consists of a group of Dementia activists who work to raise awareness of dementia at public events, co-design activities at the organisation, and contribute to, and shape the direction of, research and teaching. Members include people living with dementia, current care partners (someone currently caring for a spouse with dementia) and former care partners (someone whose family member has passed away from dementia).

Phase one: In summer 2020, (*n =* 13) semi-structured telephone interviews were conducted (SB) to better understand the participants’ perceptions of returning to in-person meetings. Interviews lasted between 13 min (FCP2) and 33 min (PLWD5) with the average interview being 22 min long.

Phase two: In the summer of 2021, following a period of virtual consultation, participants took part in the next phase of research about their experiences of returning to in-person groups. Observations of the participants return to in-person meetings were conducted (SK) at (1) an initial outdoor meeting at a local garden site and (2) their first on-site meeting at the Institute for Dementia. Mood questionnaires were also administered to capture the participants’ attitudes towards in-person re-engagement before and after their first on-site meeting (see Johnson et al., 2017). Participants were asked to rate their mood at the beginning and end of the first in-person meeting, on a scale of 1–5 (1 = Not at all happy; 5 = very happy), and a further five open questions were included so they could express their perceptions, and experiences, of re-engagement. Both questionnaires utilised visual analogue scales to measure their mood so as to make the questionnaire as accessible as possible and avoid burdening participants with overly complex and cognitively challenging measures.

In phase one, 16 Dementia Associates were approached by telephone and asked if they would be willing to participate in the study. Of these, 13 agreed to take part. In phase two, all those who decided to attend the first in-person meetings (*n* = 10) agreed to be involved in the research. Across both phases of research, 11 participants were female and three were male. The age range for participants was 50–87 years (average age 68.5 years). Five participants were living with dementia (two male and three female); two were current care partners (one female and one male); and seven were former care partners (all female). For an overview see Table 1:

**Table 1.** Participants.

Phase 1	Phase 2	Type of participant	Participant number	Gender
√	√	Person living with dementia	PLWD1	Male
√		Person living with dementia	PLWD2	Male
√	√	Person living with dementia	PLWD3	Female
√	√	Person living with dementia	PLWD4	Female
√		Person living with dementia	PLWD5	Female
√	√	Care partner	CP1	Female
√	√	Care partner	CP2	Male
√		Former care partner	FCP1	Female
√	√	Former care partner	FCP2	Female
√		Former care partner	FCP3	Female
√	√	Former care partner	FCP4	Female
√	√	Former care partner	FCP5	Female
√	√	Former care partner	FCP6	Female
	√	Former care partner	FCP7	Female

## Analysis

The datasets were analysed thematically. Thematic analysis (TA) is a widely used method of identifying and analysing patterns of meaning in a data set (Braun & Clarke, 2006). An inductive approach to analysis was employed in that no prescribed codes or themes were prepared prior to the analysis of the data. Instead, after transcription, the researchers read, reread, and coded the data corpus, and themes were conceptualised based on the data/research question and interpretively shaped by the researchers various social and cultural positionings. The two research questions specifically addressed were:

### Phase 1

How do people living with dementia and their care partners feel about re-engaging with community groups once the current lockdown restrictions are eased and what measures are required to support individuals to feel safe enough to reengage?

### Phase 2

What are the views and experiences of people living with dementia and their care partners about re-engaging with a community-based group post-lockdown?

The flexibility of this approach allowed for the necessary broad exploration of how participants felt about (1) re-engaging in community groups before they were able to reopen and (2) the specific measures that group facilitators can put in place to make the environment as safe as possible. Observational data collected during phase 2 enabled a richer understanding of how participants experienced the shift to in-person meetings. Participants were consulted about the analysis of phase 1 and phase 2 data sets. Online meetings were held with the specific purpose of deliberating the findings, and in these meetings, each thematic area was discussed, refined as required and agreed.

## Findings

Three over-arching themes were generated from the data about how individuals with dementia and care partners experienced their re-engagement with in-person community groups: (1) Planning for and the reality of transitioning; (2) Safety versus autonomy; and (3) Tensions and complexities of life in the ‘new normal’.

### Planning for and the reality of transitioning

In phase one of the study, 3–4 months after the initial national lockdown in England, participants expressed a strong desire to return to in-person meetings. Though, nuance was found in their reported willingness to reengage with groups and their opinions about safety. These differences can be most aptly described as a desire ‘to just get on’ versus a desire for a cautious approach.

As long as I can get out and meet people, I don’t care, 'cause at the moment, as I said before, to me, this is not…this is not existing. (PLWD5)

[COVID-19] is really going to have to more or less disappear before I can venture out very far. (FCP3)

Of the 13 participants at phase 1, people living with dementia and their care partners were the most eager to reengage in community groups. With the exception of PLWD1, who did not express a view, all participants with dementia and care partners appeared either keen to return to the groups immediately or to reengage in the near future, albeit with safety measures in place. Former care partners were more cautious, with the majority noting that they did not feel the groups could return safely in the near future.

It is not immediately apparent as to the difference in opinion on re-engagement, although it is possible to make some assumptions based on the content of individual phase 1 interviews. It is likely, for example, that people living with dementia and their care partners have a different lived understanding of the reality of experiencing a lockdown situation with dementia and were clearer about the negative impact this was having upon their own mental health, their wellbeing and their cognitive functioning (as reported elsewhere Bushell, Innes and Smith forthcoming). CP2 for example said that his willingness to reengage with groups immediately, stemmed from his partners need for social contact:

[The groups are] great for Annette. Different environments again, different scenery, different conversations, and activities, which is the important thing with Annette. (CP2)

While people living with dementia spoke of their willingness to reengage with community groups from the perspective of safeguarding their mental health and wellbeing, care partners and former care partners expressed some concern about how possible this might be whilst ensuring the physical safety of the group. One former care partner, for instance, questioned whether people living with dementia would have the cognitive abilities to understand social distancing guidelines in the community:

You don’t know what other individuals are going to do and what their understanding of the rules and regulations are, because unless they have that full capacity, and they know fully what it is that is being expected of them. (FCP1)

This perceived inability for people living with dementia to maintain social distance was identified as a reason why individuals might be reluctant to reengage with groups. CP1, for example, expressed some reluctance in re-engaging with others:

Because there were some people [who say] I’ve got dementia what the heck, something is going to get me anyway why worry. (CP1)

People living with dementia however felt that they could be trusted to act in line with UK Government guidelines:

Most people are sensible. Even though they might have some memory problems. (PLWD4)

Although concerns can be linked, the reality was that every individual interviewed had a different perception of the dangers of re-engaging in community groups. These ranged from a carefree attitude on one hand to extreme concern on the other. Ultimately, it was down to the individual to assess the level of risk they are willing to take once groups open up again, a point identified by CP1:

I think, we have got to decide what is acceptable risk, and we all have to live with a level of risk. What is acceptable, what is your bottom line, the things that you won't have, you know. (CP1)

However, attitudes to reengage were not solely founded upon the individuals’ perception of the threat of COVID-19. One participant living with dementia, for instance, felt that their abilities had altered to such an extent that they would struggle to attend groups:

I don’t know if I’ll be able to cope with these groups. I can't recognise faces anymore; I can’t understand names. I’m not a person for giving up, but it might be easier. Some days I think it might be easier just to chuck it all in. (PLWD3)

It is against this backdrop of concerns and their entwined desire to meet again that the planning for a return to in-person meetings was based up-on. We now consider the participants experiences of meeting in-person again.

Planning for the reality of the transition to in-person meetings required an understanding of individual readiness (on not) to engage with community groups and the reasons for their position to ensure that appropriate methods of engagement were found and that were as safe as possible. A key step in this transition to ‘on-site’ in-person meetings was having an initial meeting at a local garden at phase 2 of the study. This ‘staggered’ approach to re-engagement was designed with the participants over the course of lockdown to ensure that their voices and lived experience were integral to planning and decision-making.

In phase two of the study, when participants began to meet in-person again, they demonstrated adaptability, flexibility and an acceptance to the everyday changes they were faced with. Often, this was grounded in their understanding that being together again physically required a compromise.

People have different feelings about it all, but you just figure it out between you don’t you. It’s about respect. (CP1)

This was inherently tied to their effort to preserve the group. Where the pandemic had revealed certain divisions in how they felt about the return to – and future of – the groups, they sensed throughout both phases of the study that a lack of acceptance and resilience could mark the end of their time together:

If we’re going to keep it going, there’s no use wishing like it was. (FCP4)

Participants had a shared understanding that things will not just go ‘back to normal’; that the groups will not fall back into the form they once held. Though this was not always regarded as a source of anxiety or concern, rather also expressed as a positive in how it has given them an opportunity for change:

It’s like we’re back starting afresh, back to the drawing board really isn’t it. (FCP6)

During phase one, participants had expressed concerns around the problematic nature of reintroducing face-to-face meetings after a long period/s of isolation, but at phase two they all expressed that the transition was well-paced and stress free. Importantly, this ‘smooth transition’ was grounded in how the participants were an integral part of the planning process:

The group highlight that knowing what to expect is critical and CP1 notes a smoother transition was possible because they’ve ‘planned it for so long’. (First meeting observation)

Virtual meetings, email contact and phone calls during lockdown(s) were found to enable participants to remain involved in decision-making about the planned return. They promoted feelings of worth and autonomy and helped to identify a phased return that reflected not only COVID specific guidelines, but aligned with their own individual understandings of safety.

I like the slow return, I’m not as nervous, it’s like we are easing into reality again. (PLWD4)

When the groups did begin again participants expressed great delight in being together again, and sharing an activity outdoors as the first step to re-engagement was particularly enjoyed:

The group, who are in the herb gardens, discuss how nice it is being able to explore something new together outdoors. How being isolated makes being outside and the ability to see new things more important whilst also feel safe and not confined. (Garden observation)

During phase two, all participants noted that virtual contact had kept them meaningfully connected across the lockdowns, but it was not understood to be the same as connecting in-person, and, as such, highlighted the crucial importance of face-to-face interaction. This was particularly felt among by those living with dementia and current care partners, which reinforces the notion that loneliness has been exacerbated by the effects of the pandemic among this group:

It did the trick but wasn’t a cure - you’re still terribly isolated, especially if you have dementia. (CP1)

It’s the, ‘being together, that matters’ (PLWD1-mood questionnaire)

Obtaining the views of participants prior to returning to in-person meetings were thus key to planning the return to meeting in-person. This eased the transition for participants and responded to their need for social contact and interaction in a setting where they had preserved a keen sense of belonging and ownership.

### Safety versus autonomy

Every participant mentioned ‘safety’ in the context of mitigating the risk of contracting COVID-19. However, what ‘safety’ might entail varied widely from person to person, with those who felt happy to reengage with community groups immediately being more relaxed about safety measures.

At phase one of the study, when asked about the specific measures that might be put in the venue to make engagement in groups as safe as possible, participants mentioned the layout of the indoor space, keeping two metres apart, the outside space, the use of face masks, the toilet, the kitchen area and sharing equipment. Again, the measures they felt would be expedient varied from person to person and were impacted by their own attitude to risk and their own personal circumstances.

England’s government guidance stated that individuals from different households should stay two metres apart, and whilst some individuals felt that it was possible to achieve this within the venue, others felt it would not be possible as the venue was too small to enable any form of social distancing:

It’s quite big inside as well, and we’ve got tables and chairs, so we can keep two metres or a metre apart. (CP2)

So, the thing that bothers me is to enable us to have enough space, so that we're in the guidelines, you know, distancing. (FCP6)

Two people suggested that this might be remedied by reducing the size of the groups, while one individual living with dementia feeling that social distancing was irrelevant:

I wouldn’t mind if I was sitting right next to somebody. (PLWD5)

The outdoor space at the venue was identified by six participants as a way that groups might be able to take place while maintaining social distancing. For example:

If we’re meeting outside… to have a few tables outside of the wall, that would be good… So, I would quite like to see a few activities that we can do outside. (PLWD3)

However, it was recognised that outside groups may not be as pleasant in colder, wetter weather. In addition to this, the use of the toilet and kitchen area was raised as problematic as these facilities would still need to be open to participants even if the group chose to meet outdoors:

What my concern is the fact that we only have one toilet so if people use that toilet who’s actually going to sanitise it? Who’s going to clean it? Who has that responsibility to do that? (FCP1)

The kitchen proved a less contentious issue since it was easier to avoid using this area and for people to bring their own refreshment to groups, though sharing items was still often voiced as a concern; particularly in reference to sharing art and craft materials:

Why can’t people bring their own, like a small picnic effect, and everybody has their own drinks. (CP2)

Well, you won’t be able to like pass crafts, you know, if you’re doing crafts, everybody touches things, would you. (PLWD4)

While participants raised issues, they were not seen as unsurmountable barriers to re-engagement. The compulsory wearing of face coverings, however, was a more controversial topic amongst individuals. Individuals who appeared more risk averse during phase 1 interviews felt that coverings should be worn, however, some voiced that they did not want to wear a mask at social groups at both phase one and two of the study:

I’m not sitting there wearing a mask, no. (PLWD4)

Despite the safety measures suggested by individuals, two former care partners questioned whether it would be possible to reengage people at the usual venue at all, even if all the measures advocated were put in place:

Is it really possible under these sorts of constraints to reopen something like the [venue]? (FCP1)

Though most phase one participants (*n* = 13) appeared confident that groups could reopen successfully at some point the future. And when the groups did reopen, at phase two, participants felt confident in what was happening; mood questionnaires highlighted that all participants (*n* = 10) felt well-informed about the process of re-engagement. Although some changes were confusing, they expressed that the smaller numbers and high levels of involvement in decision making about what in-person meeting would be like had led to a calm and positive experience.

All participants generally reported feeling safe and supported to return to in-person meetings, though interestingly, at phase two of the study, care partners and former care partners showed no anxiety about group members forgetting to wear a mask or socially distance. For those living with dementia however, their ability – as well as the ability of others – to remember rules and regulations could be a source of concern:

I’m concerned about people forgetting what the rules are or being expected to remember the rules myself. (PLWD3-mood questionnaire)

This was found to be particularly difficult in how these rules were not a constant, rather shifted and changed over time and across different spaces. Keeping up with conflicting guidance was therefore felt to be cognitively taxing for participants:

The group talk about how rules are especially confusing when other groups they attend don’t follow the same rules. FCP5 notes how much uncertainty and confusion this causes. (First meeting observation)

However, alongside these concerns, those with dementia often recognised that smaller groups made it safe for them to participate with more ease, given they could follow and participate in group discussions:

I can follow what’s going on now. (PLWD1-mood questionnaire)

Participants also reported enjoying the increased level of planning that went into reformulating the groups and activities. This was expressed to not only allowed participants to feel safe, but also to allow them to claim more autonomy in a wider context that had curtailed their decision-making abilities.

I do like that we plan things more now it helps you know what’s going on, I think. (PLWD4)

Though a tension was found in the balance between participants feeling both safe in the environment at the same time as being able to maintain their independence. There was an acknowledgement among the group that the increased rules and regulations of COVID-19 can jeopardise their role in the process of planning a ‘safe return’ and therefore curtail their autonomy. Experiences of disempowerment were often discussed in relation to their inability to safely engage in everyday tasks associated with attending the groups:

It is hard, we’re part of the process yes, but we can’t just get up and make a cup of tea together. (FCP6)

The group talk about an imbalance in ‘having a say’, and that they feel they are being told what they can and can’t do. (First meeting observation notes)

While this tension was more generally felt among all participants, there was some differences apparent in how they perceived the impact of the safety measures that had been implemented. Former care partners, in particular, often noted at phase two that the on-site environment was ‘not quite the same’ or ‘missing that sparkle’. (PLWD4).

Those living with dementia and their care partners, however, reported that ‘it’s like it always was’ (PLWD3). Though, it should be noted here that amidst these differences, all participants felt that complications regarding their experience of safety measures were worth it, indicating that their return and presence there is about more than just autonomy, rather about their collective enjoyment and engagement:

There’s still an overarching sense of happiness though [...] the participants discuss the positive difference that seeing each other face to face - albeit briefly every week - makes. (Second meeting observation)

The tensions in providing a safe environment while also preserving and rebuilding autonomy is complex and underpins some of the broader issues we now consider in relation to the ‘new normal’.

### Tensions and complexities of life in the ‘new normal’

Whilst participants had look forward to re-engaging with the groups in person, they recognised that the nature of group activities would have to change significantly when these meetings were permitted. Art, craft and gardening activities, for example, were seen as challenging:

If you’re inside and you’re running a group, say like an art group, well how can you do that? Because people are touching different things that other people have had. (FCP1)

One individual living with dementia spoke of their regret that the groups would have to look and feel different to adhere to Government and University guidelines and mitigate the risk of spreading COVID-19:

I know it’s not going to be like it was, and that’s another thing that really upsets me. (PLWD5)

While another highlighted the fact that people living with dementia may struggle to come to terms with those changes both in community groups and community settings more broadly:

I suppose the way we do things now is so different and it might be difficult for people maybe living with dementia to adapt as quickly as that. (PLWD2)

A common understanding thus existed amongst participants that careful consideration should be paid to the reality of engagement post-lockdown(s). In this way, whilst all participants reported via both the first and second mood questionnaire that they felt happy and well supported to return to meet in-person, data during phase two highlighted key changes in how they experienced this space. Some participants felt they the environment was safer than ever; somewhere they felt comfortable and content:

Nice to be back it feels like a safe warm space. (PLWD4 – mood questionnaire)

I feel safer than ever and happy to return (FCP5 – mood questionnaire)

However, the participants’ experience of the environment was multifaceted. The space also represented one of loss. With reduced numbers at the groups given regulations around distancing, some group members choosing not to return, and a number of deaths within the group over the past 2 years, the participants felt these social changes had shifted their relationship with the venue:

The participants look at the photos of past group members of the wall and talk about how different it feels there with the loss of those who have left or died. FCP6 says: ‘it’s just not the same place without them’. (Second meeting observation)

Further, while the participants in the moment experiences often reflected feelings of satisfaction once they returned, it became apparent at phase two that the uncertainty of the pandemic continued to unsettle their perceptions about the *future* of this environment. For instance, CP1 questioned:

Will it (the venue) ever be the same as before, we just don’t know. (CP1)

The pandemic thus led the group to reconceptualise their relationship to the group venue in complex ways, and, as such, could result in them feeling both emplaced and displaced all at once. Related to this was the complexity the participants experienced when navigating the physical space of the venue. The first outdoor meeting gave them room to negotiate the environment with ease, and move through it in ways that felt right to them, though certain difficulties became apparent at the first indoor venue meeting. While some participants quickly aligned with social distancing measures and mask wearing regulations, others showed a gentle resistance in effort to connect in a way that they wanted and therefore reminders to adhere to regulations were necessary.

There are some reminders needed to separate and distance – there’s difficultly in balancing the experiences and perceptions of different people. (Second meeting observation)

Although these reminders were not met with hostility, they could disrupt the flow of the atmosphere among the group and set the scene for bursts of interactional tension. In addition, more generally, the practicalities of everyday movements – such as leaning in to listen to a story and going to the toilet – could also prove difficult; and a new focus on them could result in a challenge to keep the space feeling naturalistic and familiar.

Importantly though, this struggle to maintain a sense of independence was experienced differently by those living with dementia and care partners/former care partners. Where those living with dementia expressed that the ‘slower paced’ environment at their first in-person meet was empowering in how it allowed them to participate without feeling overwhelmed, care partners and former care partners felt the activities were too simplistic and thus *dis*empowering as they were not challenging enough to suit their needs:

Group unanimous that today has been special and enjoyed there not being ‘too much’ on. PLWD3 looks forward to hosting the next group activities and notes it gives them a purpose again. (First meeting observation)

Former care partners, mention that the activities are ‘nice’ but it would have been good to have a bit more ‘going on’ moving forwards. This is a different response than the individuals living with dementia, who felt that the slower pace of crafting was a gentle way to reconnect and not too overwhelming. (Second meeting observation)

This balance between the empowerment and disempowerment of participants as they resumed attending in-person meetings was tied to their broader concern to advance and contribute to the ‘dementia agenda’ for change, awareness and improvement. As members of the Dementia Associate Panel, they had previously enjoyed experiencing collective and individual influence by sharing their views and experiences pre lockdown (Innes et al., 2021). This was spoken about something they wished to pick up again, though with the pandemic bringing life to a standstill, the stagnant nature of the world was often raised as an underlying source of tension given the impact it had on their ability to plan and push the agenda forwards. Participants often expressed that they had lost their purpose and place:

I just don’t feel useful anymore, you want to make a difference more than ever now and we can’t really, can we. (FCP5)

This begins to highlight how being part of the group post lockdown is not just about socialising for the participants, rather about how it empowers them to come together in a supportive, common space to advocate and make a difference at a time where it is ‘needed more than ever’. (CP1 – mood questionnaire) It should be noted however that participants did not passively accept barriers to activism, rather they wanted to find ways to work around them and reported looking forward to the challenges of finding new ways to approach their engagement and action.

Group are discussing the garden party planned in September. Who to invite, what difference can be made, getting the message out there. Seem ready to be in the driving seat again. PLWD3 notes: ‘you need to feel like you’re making a difference to something’. (First meeting observation)

Their effort to do this was tied to a re-evaluation of their involvement in the group. Where the participants could, at times, feel they had lost their purpose because their sense of activism was being curtailed by the pandemic, their desire to regain this was more important than ever as they felt periods of isolation had led them to reimagine and review their lives and commitments.

## Discussion

This study contributes to the identified gap in knowledge about the social impact of the COVID-19 pandemic on people living with dementia and their care partners (Greenberg et al., 2020). In doing so, it demonstrates three important issues. First, we establish that the involvement of participants in ongoing discussions about what a return to in-person meeting would/should look like throughout lockdown enables a joint process to be developed, which in turn allows all participants to feel valued, safe, and consulted about their re-engagement. This was of great significance to the participants given a co-creation approach corresponded to the ethos of the groups they had previously attended at the dementia institute (Bowker et al, 2020). Although, it should be recognised that experiences of disempowerment in both experiencing the physical space itself and the modes of engagement available spoke to (1) a complexity in ensuring these values are promoted in the landscape of a pandemic and (2) the centrality of working to ensure autonomy is respected. Second, we highlight that while all categories of participants wished to return to a physical place that they had enjoyed meeting at previously, people living with dementia and current care partners expressed different views than former care partners on what re-engagement should look like and the significance of meeting in-person again (Rochford-Brennan & Keogh, 2020). Third, and building on this, such findings are of importance as they indicate that groups should not only strive to be inclusive and empowering but must also *evolve* to enable the groups to meet their varied needs and preferences. We draw particular attention to the changing nature of the group members needs and preferences during the pandemic; when former care partners returned to the venue, they wanted a ‘livelier’ feel to the meetings in order to maintain their activism, whereas the perceptions of people living with dementia shifted and they valued the slower pace to adjust to their return to this ‘familiar yet strange’ place. This research therefore brought out stark differences in the priorities of different groups according to their care giving status, and reflects the concerns raised at the beginning of the pandemic about the differential impact on different groups (Korczyn, 2020). As the British Geriatrics Society (2020) highlight, knowledge of the experience of older people during *and* after lockdown restrictions is important and this paper therefore makes a small, but important, contribution to the evidence base on transitioning to the ‘new normal’ of groups meeting again after protracted periods of physical and social isolation.

## Limitations

This research was based on a small sample of participants from a geographically distinct existing cohort of group members who were known to the researchers through previous community engagement and research work. Consequently, there are many others experiences of dementia and lockdown that we were unable to include. Given the researchers pre-existing relationships with the participants, we also recognise that individuals might have felt obliged to participate. To address this, it was made clear throughout that the participants did not have to take part in either phase of the research.

## Conclusions

Given our understanding of the way individuals re-engage with community groups post lockdown has significant impacts for the well-being and quality of life of individuals with dementia and their care partners, this research has important implications for such groups moving forward. In demonstrating the importance of consulting with, and being informed by, group members regarding their perceptions of returning to in-person groups, the study highlights the importance of ensuring re-engagement is a process designed *together* with people living with dementia and their care partners. In this way, we emphasise that it is important to find the balance between ensuring individuals feel supported and safe, whilst also respecting that their perceptions of risk – and consequently to re-engagement – are varied and should be considered when reinstating groups. While shared decision-making and open on-going discussions about perceptions of safety and inclusion contributed to a successful transition to meeting in-person again for our participants, restrictions to their autonomy were felt to curtail their involvement in advocating for dementia awareness and rights (Bowker et al, 2020; Innes et al., 2021). Such findings reflect the broader concern of lobbying groups in the UK and across the globe who fear that the COVID-19 pandemic – and the resultant loss of funding, priority, and sense of urgency in light of other health priorities – might be indicative of a ‘step back’. We therefore highlight that whilst a pandemic can pose difficulties in promoting a participatory approach in this context, importance must be placed on ensuring those living with dementia and their care partners are still enabled and empowered to achieve a sense of purpose and meaning and that their voice continues to be heard. This study provides an example of how successful re-engagement was achieved, and also points to the need for further research focused on the complex nature of returning to in-person social contact for individuals living with dementia and their care partners.
